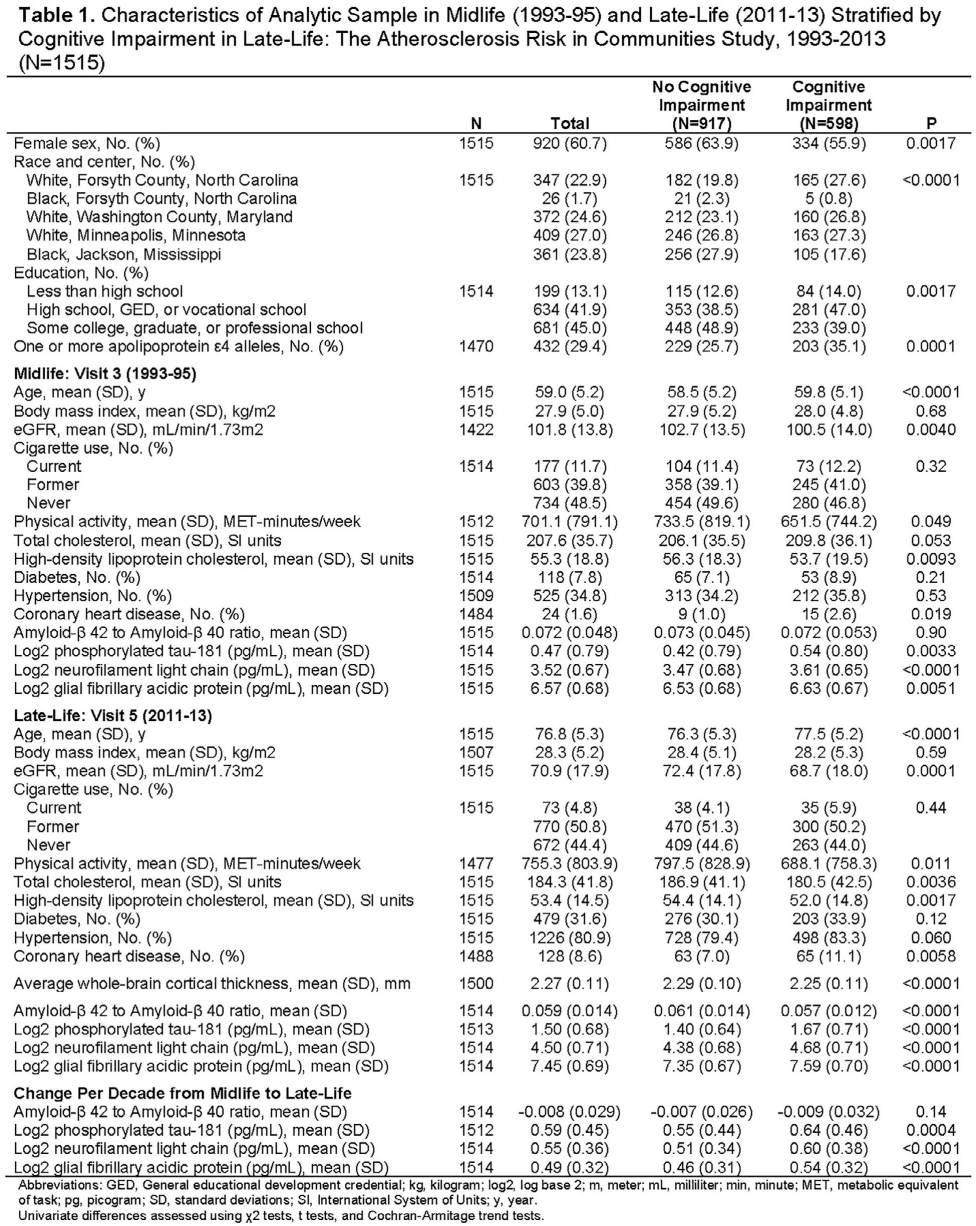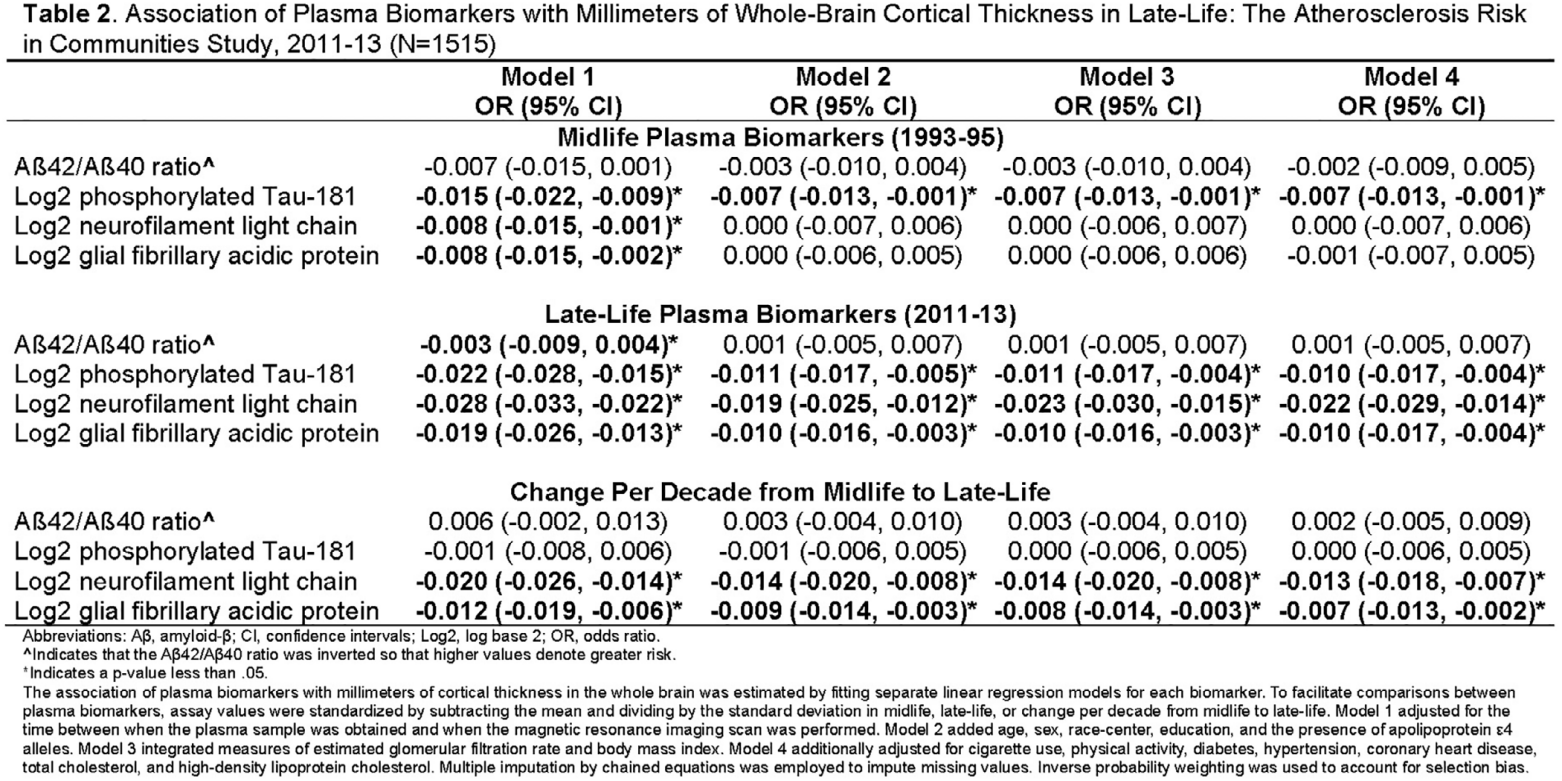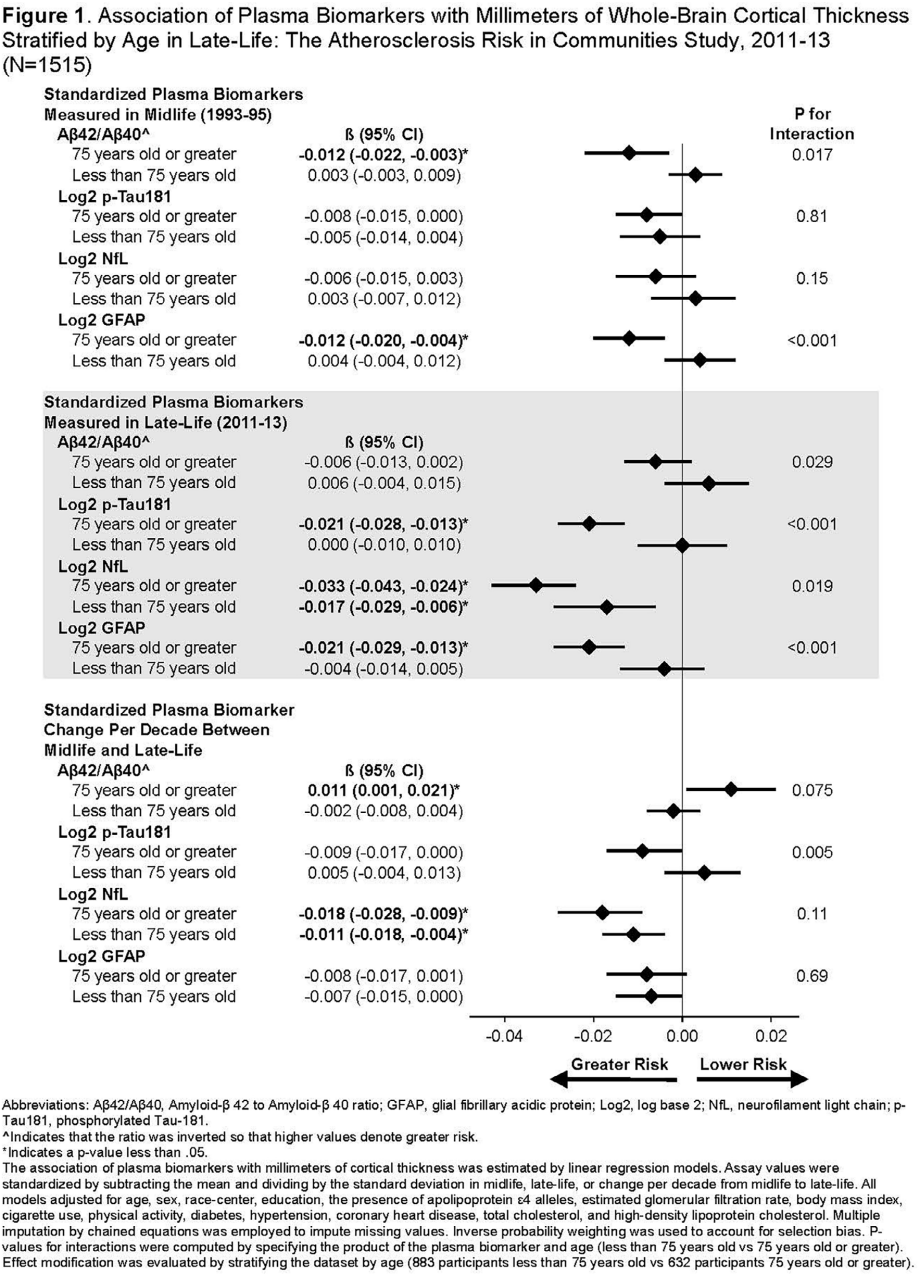# Mid‐ to Late‐Life Changes in Plasma Biomarkers of Alzheimer's Disease Pathology and Neurodegeneration and Associations with Cortical Thickness: The Atherosclerosis Risk in Communities Study

**DOI:** 10.1002/alz70856_106603

**Published:** 2026-01-08

**Authors:** James Russell Pike, Yifei Lu, Keenan A. Walker, Kevin J. Sullivan, Michael E. Griswold, Bharat Thyagarajan, Michelle M Mielke, Pamela L. Lutsey, Timothy M. Hughes, David S. Knopman, A. Richey Sharrett, Thomas H. Mosley, Rebecca F. Gottesman, Clifford R. Jack, Josef Coresh, Priya Palta

**Affiliations:** ^1^ Departments of Population Health and Medicine, New York University Grossman School of Medicine, New York, NY, USA; ^2^ University of North Carolina Chapel Hill, Chapel Hill, NC, USA; ^3^ National Institute of Aging Intramural Research Program, National Institutes of Health, Bethesda, MD, USA; ^4^ University of Mississippi Medical Center, The MIND Center, Jackson, MS, USA; ^5^ University of Minnesota, Minneapolis, MN, USA; ^6^ Division of Public Health Sciences, Wake Forest University, School of Medicine, Winston‐Salem, NC, USA; ^7^ University of Minnesota School of Public Health, Minneapolis, MN, USA; ^8^ Wake Forest University School of Medicine, Winston‐Salem, NC, USA; ^9^ Mayo Clinic, Rochester, MN, USA; ^10^ Johns Hopkins University Bloomberg School of Public Health, Baltimore, MD, USA; ^11^ National Institute of Neurological Disorders & Stroke, Bethesda, MD, USA; ^12^ University of North Carolina at Chapel Hill, Chapel Hill, NC, USA

## Abstract

**Background:**

Plasma biomarkers show promise as a noninvasive method for measuring Alzheimer's disease pathology and neurodegeneration throughout the lifecourse. However, the relationship between longitudinal changes in these biomarkers and late‐life brain morphology in community‐dwelling populations requires further investigation.

**Method:**

Between 2011 and 2013, 1,977 participants from the Atherosclerosis Risk in Communities Study received an adjudicated cognitive diagnosis and underwent 3 Tesla magnetic resonance imaging scans. Whole‐brain cortical thickness was quantified by Freesurfer. Stored plasma samples collected in midlife (1993‐95, mean age 59.0 years) and late‐life (2011‐13, mean age 76.8 years) from a subsample of 1,515 participants were assayed in 2022 using Quanterix SiMoA. The assay quantified amyloid‐β (Aβ)42/40, phosphorylated tau at threonine 181 (*p*‐Tau181), neurofilament light (NfL), and glial fibrillary acidic protein (GFAP). Linear regression models estimated the association of plasma biomarkers from midlife, late‐life, and the change from midlife to late‐life with cortical thickness in late‐life. Interactions and effect modification by age were examined in exploratory analyses.

**Result:**

The sample (Table 1) included 920 (60.7%) women, 387 (25.5%) Black participants, 513 (33.9%) participants with mild cognitive impairment, and 85 (5.6%) participants with dementia. The average age in late‐life was 76.8 (5.3 SD) years and the average cortical thickness was 2.27 (0.11 SD) millimeters. Cortical thickness was lower in individuals with adjudicated mild cognitive impairment or dementia compared to individuals without cognitive impairment (2.25 mm versus 2.29 mm, *p* <0.0001). In models adjusted for demographics, lifestyle factors, cardiovascular factors, and the presence of APOE ε4 alleles, higher midlife measures of *p*‐Tau181 and increases from midlife to late‐life in NfL and GFAP were associated with lower cortical thickness in late‐life (Table 2). Concurrent measures of plasma biomarkers in late‐life were associated with lower cortical thickness (Table 2) and exhibited a stronger association among participants ≥75 years compared to <75 years (Table 3).

**Conclusion:**

Plasma biomarkers of Alzheimer's disease pathology and neurodegeneration obtained in midlife and late‐life were associated with late‐life differences in cortical thickness. Additional longitudinal research is needed to determine whether plasma biomarkers predict cortical thinning over time and may serve as an early indicator of accelerated brain atrophy.